# Quantification of Sensitization in Aluminum–Magnesium Alloys Through Frequency-Dependent Ultrasonic Attenuation

**DOI:** 10.3390/s25133983

**Published:** 2025-06-26

**Authors:** Songwei Wang, Haiying Huang

**Affiliations:** Department of Mechanical and Aerospace Engineering, University of Texas at Arlington, 500 W. First Street, Arlington, TX 76010, USA; songwei.wang@mavs.uta.edu

**Keywords:** Aluminum–Magnesium alloy, frequency-dependent attenuation, laser ultrasonic testing, material characterization, non-destructive testing, sensitization, ultrasonic attenuation

## Abstract

Aluminum–Magnesium (Al–Mg) alloys undergo sensitization, i.e., the precipitations of β-phase (Al_2_Mg_3_) at the grain boundaries, when exposed to elevated temperature. This microstructural change increases the susceptibility of Al–Mg alloys to intergranular corrosion, exfoliation, and stress corrosion cracking. This study introduces a time-frequency analysis (TFA) technique to determine the frequency-dependent ultrasonic attenuation parameter and correlate the frequency-attenuation slope to the Degree of Sensitization (DoS) developed in heat-treated Al–Mg alloy samples. Broadband pitch-catch signal was generated using a laser ultrasonic testing (LUT) system, from which the narrowband pitch-catch signal at different frequencies can be digitally generated. The attenuation parameters of sensitized Al–Mg samples were determined from these narrowband pitch-catch signals using the primary pulse-first echo (PP-FE) method. By identifying the frequency range within which the attenuation parameter is linearly proportional to the frequency, the slopes of the frequency-attenuation relationship were determined and correlated with the DoS values of the sample plates. The experimental results validate that the frequency-attenuation slope has a higher sensitivity and lower scattering as compared to other conventional ultrasonic attenuation measurement techniques.

## 1. Introduction

Al–Mg alloys are widely used in aerospace, petroleum, and shipbuilding due to their corrosion resistance [1,2,3]. However, when exposed to elevated temperatures, these alloys undergo sensitization, where β-phase (Al_2_Mg_3_) precipitates at grain boundaries. This microstructural change increases susceptibility to intergranular corrosion, exfoliation, and stress corrosion cracking, compromising structural integrity and service life [4].

The standard method for quantifying sensitization is the nitric acid mass loss test (NAMLT), defined in ASTM G67 [5]. It provides a quantified parameter, known as the DoS, for assessing corrosion susceptibility. Although reliable, NAMLT is destructive, time-consuming, and limited to prepared laboratory samples. Other methods, such as metallography [6,7], eddy current testing [8,9], or on-site DoS probe [10,11], either only characterize sensitization at the surface or have limited depth penetration. The requirement for sample and surface preparation makes them costly and impractical for widespread use. A technique that can probe the interior of the material may be more sensitive than surface-based techniques because it allows the signal to interact with the material over a larger volume. However, as sensitization can vary locally due to grain size and composition [12,13], a faster and non-destructive technique that can take measurements rapidly at multiple locations is needed.

Researchers have explored ultrasonic testing (UT) for sensitization characterization. Unlike metallography and electrochemical techniques, UT enables large-volume assessment and subsurface characterization. Additionally, UT offers a high signal-to-noise ratio and is adaptable for in situ inspections [14]. These UT techniques can be categorized as the time-domain (TD) and frequency-domain (FD) method. The earliest TD UT sensitization study, to the best knowledge of the authors, was conducted by Li et al., who employed electromagnetic acoustic transducers to measure ultrasonic attenuation in as-received and sensitized AA5083 samples [15]. This study generated longitudinal and shear waves and detected the reflections from the sample’s backside, i.e., the pulse-echo signals. The ultrasonic attenuation parameter was determined by fitting an exponential function to the peak amplitudes of the first 4–5 successive echoes. Their results indicated that the shear attenuation parameter decreased by 10% at two frequencies, i.e., 1.5 MHz and 3 MHz. However, the large sample-to-sample variations in the shear attenuation parameter renders the study inconclusive. Chukwunonye et al. investigated the pulse-echo method using piezo-transducers to characterize sensitization in AA5083 and AA5456 samples [16]. Their study confirmed Li’s findings that the velocity of the ultrasound waves changes very little with sensitization. However, the change in the longitudinal wave attenuation was up to 20% for one AA5456 sample subjected to 120 h of heat treatment at 175 °C. Since their method is identical to that of Li’s study, except that they used a different type of transducer, the reason for this reported improvement is not clear and was not discussed. Additionally, this study calculated the measurement uncertainty from repeated measurements on one single sample. Thus, it did not account for the sample-to-sample variation as reported in Li’s paper. The curve-fitting method used in these two studies requires a minimum number of usable pulses, which limits the method’s application to materials with low ultrasound loss. Stella et al. used only the first two consecutive pulses to calculate ultrasound attenuation in austenitic stainless steels for sensitization characterization [17]. However, this two-peak method has not been studied for sensitization characterization of Al–Mg alloys.

FD ultrasound attenuation methods typically calculate attenuation from the spectral ratio of two pulses—either by comparing the same pulse at different states [18] or two consecutive pulses in a single signal [19,20]. These methods allow for determining the attenuation spectrum, i.e., the frequency-attenuation relationship. Comparing the same pulse at different states minimizes geometric or diffraction effects, while comparing two pulses in one signal provides absolute attenuation values. but it requires high signal quality and may be affected by noise or diffraction. As attenuation in alloys increases approximately linearly with frequency [21,22], the slope of the frequency-attenuation curve is also sensitive to microstructural changes. However, to the authors’ knowledge, no study has been carried out to study this parameter for sensitization detection in Al–Mg alloys.

Expanding the work of Wang et al. on sensitization characterization using LUT systems [23], this study studies the correlation between the frequency-attenuation slope and the DoS developed in heat-treated AA5456 sample plates. The LUT set-up and characterization of DoS using NAMLT are first described. The impulse responses of the sample plates, acquired using the LUT system, were analyzed in the time-frequency domain to extract the attenuation parameter at discrete frequencies. The slope of the frequency-attenuation curve was calculated and correlated with the corresponding DoS. For comparison, two conventional attenuation determination methods, i.e., the multi-pulse exponential curve-fitting method and the PP-FE method, were applied to the same sets of data. In addition, AA5052 alloy samples were tested as a reference material. The results show that the frequency-attenuation slope, obtained using the PP-FE method, offers greater sensitivity and lower variability for sensitization characterization.

## 2. Experimental Procedure

### 2.1. Material Selection, Sample Design, Heat Treatment, and NAMLT DoS Characterization

To study the influence of sensitization on the frequency-dependent attenuation α(f) of heat-treated Al–Mg alloy, three sample plates were fabricated from cold rolled AA5456, a marine grade aluminum alloy with 4.7 wt.% to 5.5 wt.% magnesium. For comparison, two plates were fabricated from cold rolled AA5052, an aluminum alloy with a lower magnesium content of 2.2 wt.% to 2.8 wt.%. These plates were heat-treated side by side in an oven at 120 °C to induce sensitization in AA5456 but not in AA5052 [24].

The plate’s thickness is 6.35 mm (1/4 inches). To ensure that the longitudinal wave can travel at least five round trips along the plate thickness before overlapping with reflections from the plate edges, the half-length and half-width of the plate should be larger than 31.75 mm. Taking the margin of measurement range and fabrication convenience into consideration, the length and width of the sample plates were selected to be 101.6 mm (4 inches). The as-received cold rolled sheet has sufficient flatness and roughness. Thus, no machining is needed for the sample surfaces. The sample plates were heat-treated at 120 °C up to 168 h, during which the heat treatment was interrupted at a pre-selected schedule so that LUT can be carried out. Initially, the AA5456 sample plates received three 8 h heat treatment increments, starting from their pristine state, and progressing to 24 h of heat exposure. Subsequently, an additional four 12 h heat treatment increments were applied, reaching a cumulative heat exposure duration of 72 h. Lastly, four 24 h heat treatment increments were conducted, totaling 168 h of heat exposure. The gradually increasing heat treatment increments were selected based on the nonlinear progression of the sensitization process, which initially progresses rapidly and then gradually slows down. Employing smaller initial heat treatment increments enabled us to closely monitor the sensitization progression at early stages. The reference AA5052 samples underwent three heat treatments, 24 h, 72 h, and 120 h.

**Figure 1:**
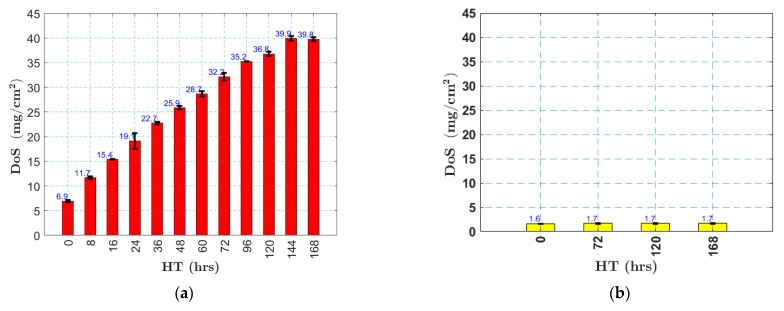
Changes in the DoS values of (**a**) AA5456; (**b**) AA5052, heat treated at 120 °C, measured using nitric acid mass loss test (NAMLT). The black lines indicate the variance between two match-stick samples.

To monitor the progression of sensitization, matchstick samples were machined from the as-received materials and heat-treated along with the sample plates. The matchstick samples have a length of 50 mm and a cross-section of 6.35 mm by 6.35 mm. At each heat treatment interval, two matchstick samples were removed from the oven, and their DoS was characterized following the ASTM standard G67 [5]. The mass of the matchstick samples was first measured using a digital scale with a precision of 0.1 mg. The samples were then immersed in a 70 wt.% nitric acid solution at 30 °C for 24 h and their mass was measured again to calculate the DoS, i.e., the mass loss per unit area (mg/cm^2^). Figure 1 shows the NAMLT results obtained from both AA5456 and AA5052 samples. The DoS of AA5456 samples increased with heat treatment durations monotonously, as shown in Figure 1a. The pristine AA5456 samples had a DoS of 6.9 mg/cm^2^. The DoS increased rapidly to 19 mg/cm^2^ after 24 h of HT, but the increase rate reduced afterwards; the DoS reached 36.8 mg/cm^2^ after 120 h of HT, and finally saturating at 39.9 mg/cm^2^ after 144 h of HT. In contrast, the averaged DoS of the pristine AA5052 samples was only 1.7 mg/cm^2^ and the DoS did not change much with HT, validating that AA5052 is not susceptible to sensitization, as shown in Figure 1b. The error bars in Figure 1a,b were calculated from the DoS values of two matchstick samples underwent the same HT. The small error bars indicate that the two matchstick samples have relatively consistent DoS values, especially for AA5052.

### 2.2. Laser Ultrasonic Testing

**Figure 2:**
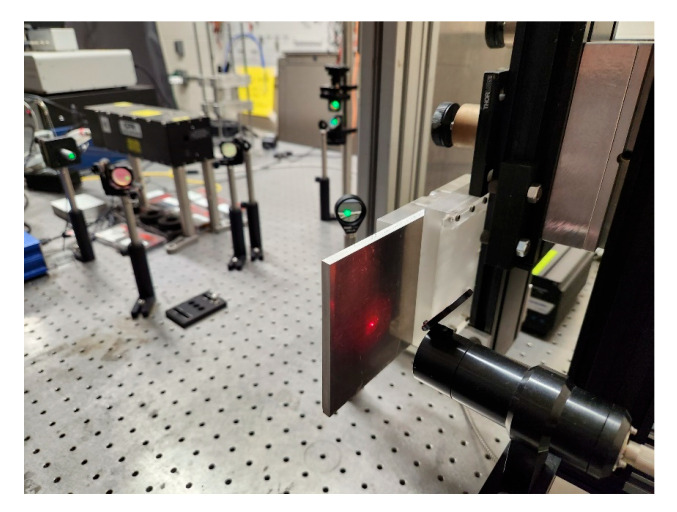
Measuring the impulse responses of a sample plate using a Laser Ultrasonic Test (LUT) system in B-scan mode.

The impulse responses of the sample plates were measured using an LUT system from Intelligent Optical Systems (IOS) in B-scan mode, as shown in Figure 2. The excitation laser was a Q-switched free space Nd:YAG pulse laser (Quantel ICE 450 with Ultra laser head, Quantel laser by lumibird, Bozeman, MT, USA), positioned to the left side of the sample plate. It has a wavelength of 1064 nm and a peak energy of 200 mJ. The energy of the laser pulse was limited to 12% of the total energy, which was controlled by the laser control software (AtCalibration 5.1.2). The pulse width of the excitation laser is 10 ns at a repetition rate of 5 Hz. Using a spherical lens with a 200 mm focal length, the focus of the excitation laser was adjusted to be just above the abrasion limit of the material, i.e., it leaves a barely visible scar on the sample surface. Under this mode, the laser-generated ultrasound guided wave is dominated by the longitudinal wave that propagates normally to the sample surface [25]. The out-of-plane displacement of the sample plates was measured near the irradiation point at the opposite side of irradiation using an adaptive two-wave mixing laser interferometer. The sensing laser was a 1550 nm continuous wave fiber laser with a 2 W peak power, connected to a variable power splitter that outputs a probe beam and a reference beam (IOS AIR-1550-TWM Laser Ultrasonic Inspection System). The probe beam is directed at the sample surface and its reflection is collected by a fiber bundle. These two beams interfere at an angle in a photorefractive crystal to produce a real-time interference hologram, converting the phase change of the reflected beam, induced by the surface displacement of the sample, into an amplitude change [26]. During this research, attenuation was observed to increase approximately linearly with frequency across the measured range; this linear relationship was most pronounced between 10 and 16 MHz. To improve signal strength in the 10–16 MHz band, the interferometer’s variable power splitter was adjusted to boost the detected signal amplitude. This adjustment did not alter the inherent linear frequency-attenuation trend. The output signal of the laser interferometer was acquired using a National Instruments 8-bit data acquisition card (NI PCI-5114), housed in a personal computer (PC). The data acquisition process was configured and controlled using the LaserScan v2.36 provided by IOS and triggered by the sync signal generated by the excitation laser. The acquired data were filtered using a 20 MHz low-pass filter provided by the Laser-Scan software.

To measure multiple locations in a single set-up, the sample plate was mounted on a PC-controlled two-axis translation stage for B-scan inspections along a predefined line. At the start of the experiment, the excitation and sensing lasers were first aligned at a corner of the sample plate. The plate was then translated to the initial inspection point, defined as (*x*, *y*) = (0, −10) mm, which is 10 mm below the geometric center of the plate. This point served as the starting position for the B-scan. Afterward, the laser focal points were adjusted to ensure proper focus on the sample surface. The translation stage then incrementally moved the sample along the *y*-axis from −10 mm to +10 mm in 0.4 mm steps, resulting in 51 measurement points per B-scan. Since the LUT system was not placed on a vibration isolated optical table, the measurements at each location were averaged over 128 excitations.

## 3. Time-Frequency Analysis of LUT

**Figure 3:**
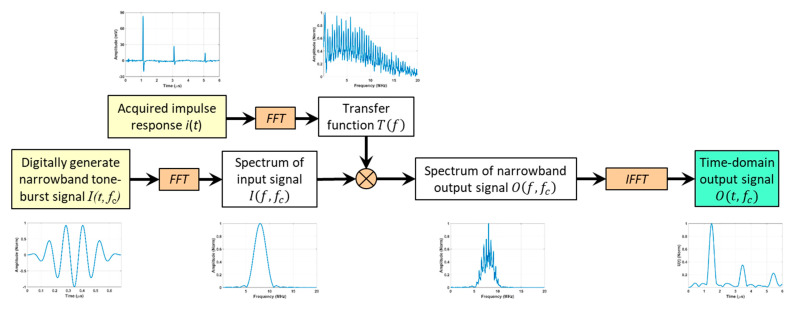
Time Frequency Analysis of broadband Laser Ultrasonic signal to extract the attenuation parameter *α*(*f*). The cross-in-circle symbol denotes multiplication. The transfer function *T*(*f*) is multiplied by the spectrum of input signal *I*(*f*, *f_c_*) to obtain the spectrum of narrowband output signal *O*(*f*, *f_c_*), from which the attenuation parameter *α*(*f_c_*) is determined using the primary pulse-first echo (PP-FE) method.

To obtain the frequency-dependent attenuation parameter *α*(*f*) from the broadband LUT, a TFA technique, is developed based on the network theory and the digital signal processing technique described in [27]. As shown in Figure 3, the acquired ultrasound signal is treated as the impulse response of the sample plate up to 20 MHz, which is the upper cut-off frequency of the LUT system set by the LaserScan v2.36. Treating the sample plate as a linear time-invariant (LTI) network, the Fourier transform (FT) of the impulse response is thus the transfer function of the sample plate *T*(*f*). In the frequency domain, the response of the sample plate subjecting to any arbitrary narrowband input with a center frequency of $f_{c}$, i.e., $I\left(f,f_{c}\right)$ can then be calculated as

(1) $$O\left(f,f_{c}\right)=T\left(f\right)I\left(f,f_{c}\right).$$

The corresponding narrowband time domain response can thus be calculated by applying the inverse fast Fourier transform (*IFFT*), i.e.,

(2) $$O(t,f_{c})=IFFT\left[O(f,f_{c})\right]=IFFT\left[T\left(f\right)I(f,f_{c})\right].$$

The narrowband tone-burst signal $I(t,\;f_{c})$, as shown in Figure 3, can be digitally generated as a tone-burst signal with an arbitrary center frequency $f_{c}$. As shown in Figure 3, the narrow band response of the sample plate $O(t,\;f_{c})$, calculated using Equations (1) and (2), has multiple peaks at the same arrival times as the impulse response. These peaks are not as sharp due to their narrow bandwidths. Assuming there is sufficient time separation between the peaks, the attenuation parameter at the tone-burst center frequency, i.e., ${\alpha (f}_{c})$, can be determined by either fitting the amplitudes of the first few peaks to the exponential decay model [15,16], i.e.,

(3) $$U_{n}=U_{P}e^{-2\alpha nl},\;n=1,\;2,\;\ldots ,$$

or from the amplitudes of the primary pulse and the first echo [17], i.e.,

(4) $$\alpha =20\log\left[\frac{U_{P}}{U_{2}}\right]/2l.$$

in which U is the amplitude of the peaks. The subscript P stands for the primary pulse and the subscript *n* stands for the *n*th pulse. In Equation (4), $U_{2}$ represents the first echo. For convenience, this method is named the primary pulse-first echo, i.e., PP-FE, method.

**Figure 4:**
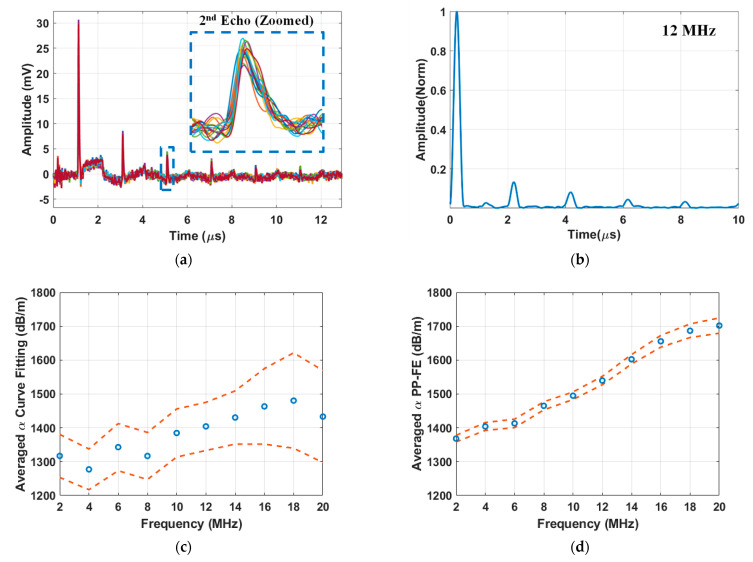
Laser ultrasound test results of AA5456 #1 after 168 h of heat treatment (DoS = 39.8 mg/cm^2^) (**a**) the broadband response of the sample plate measured at 51 B-scan locations; each different colored trace represents an individual measurement at one location; (**b**) Narrowband response of the acquired signal from a single B-scan location, calculated using the time-frequency analysis technique with a 12 MHz tone-burst input signal. Changes in the averaged B-scan attenuation parameter with the input frequency, determined using (**c**) the curve fitting method and (**d**) the PP-FE method. Dashed lines represent the upper and lower limits of the 95% confidence interval of 51 B-scan measurements.

Figure 4a presents the acquired B-scan impulse responses of AA5456 sample #1 after 168 h of heat treatment (DoS = 39.8 mg/cm^2^). Each pulse corresponds to a longitudinal wave reaching the detection side. As expected, the pulse amplitude decreases monotonously due to the round-trip attenuation. The sixth pulse, arriving at around 11 μs, is barely distinguishable with its peak amplitude just above the noise floor. The zoomed-in view of the second echo is shown in the insert of Figure 4a. The arrival time and amplitude exhibit slight variations across different B-scan locations, reflecting the influence of the spatial variability on these parameters.

The narrowband response of the sample plate, produced from the broadband LUT signal measured at one location with a 12 MHz narrowband tone-burst signal as the input, is shown in Figure 4b. The signal, normalized to the amplitude of the primary pulse, displays clearly separated but broadened peaks for attenuation determination. To account for spatial variability, the attenuation parameter was determined individually at each B-scan location using both the curve fitting and PP-FE methods. Figure 4c presents the averaged frequency-dependent attenuation parameter determined using the curve fitting method. The attenuation parameter values show an overall increase of 116.22 dB/m over the frequency range from 2 MHz to 20 MHz. The dash lines, representing the 95% confidence intervals, indicate a margin of error varying between ±30.69 dB/m and ±71.79 dB/m, reflecting significant variability at different locations. In contrast, Figure 4d illustrates the frequency-dependent attenuation parameter calculated using the PP-FE method. The PP-FE method exhibits a lower error margin of less than ±22.57 dB/m and a larger overall increase in the attenuation parameter of 333.68 dB/m. Because of its higher frequency sensitivity and reduced scattering, the PP-FE method is more suitable for studying the influence of sensitization on frequency-dependent attenuation parameters. We also attempted to use the spectral ratio method to study the frequency-attenuation relationship [19,20]. It involved time gating two consecutive pulses, calculating their spectra using FT, and then calculating the ratio of the two spectra. Although it used the same two pulses as the PP-FE method, the frequency-attenuation relationship showed larger variations compared to the present method. In addition, the selection of the time-gating window also strongly influences the resulting spectra ratio. Based on the results, we concluded that the spectral ratio method is not suitable for sensitization characterization.

## 4. Correlation of Ultrasonic Attenuation and DoS

**Figure 5:**
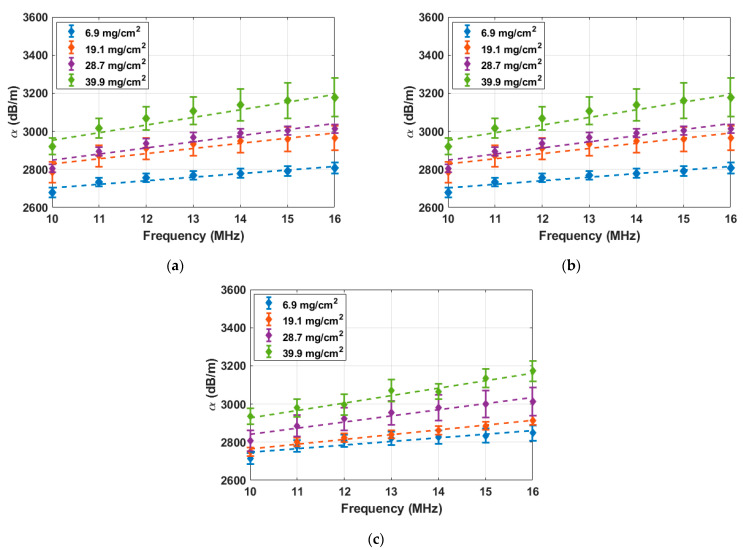
Changes in the longitudinal-wave attenuation parameter α with frequency for four selected DoS values in three AA5456 samples: (**a**) AA5456 #1, (**b**) AA5456 #2, and (**c**) AA5456 #3. Each data point represents the average attenuation value calculated from 51 B-scan measurements, with error bars indicating the 95% confidence interval. The dashed lines indicate the linear fits used to extract the frequency-attenuation slope.

Figure 5 presents the frequency-dependent attenuation parameter, calculated using the PP-FE method, for the three AA5456 samples at four different DoS values, 6.9 mg/cm^2^, 19.1 mg/cm^2^, 28.7 mg/cm^2^, and 39.9 mg/cm^2^. The corresponding heat treatment durations for these DoS values are Pristine, 24 h, 60 h, and 168 h. The attenuation parameter values are plotted over the 10 MHz to 16 MHz frequency range with 1 MHz increments. Each data point represents the average attenuation parameter calculated from 51 B-scan measurements. The error bars represent the 95% confidence intervals of the average attenuation parameter at each DoS level. Each set of frequency-dependent attenuation values was fitted using a linear regression to extract the frequency-attenuation slope. The resulting fitted lines are shown as dashed lines. Across all three samples, the attenuation parameter consistently increases with both frequency and DoS. Despite some sample-to-sample variations, the slope of the frequency-attenuation relationship consistently increases with DoS.

**Figure 6:**
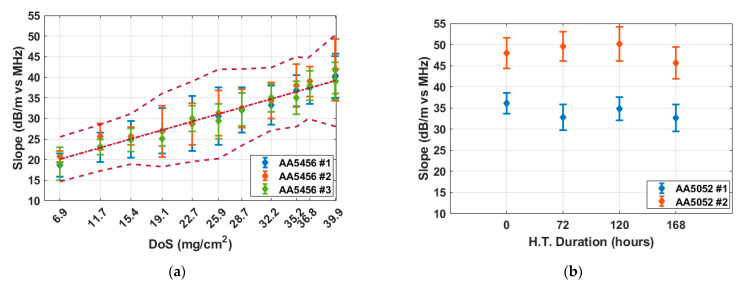
Averaged slopes of frequency-attenuation relationship as a function of DoS for (**a**) AA5456; (**b**) AA5052 samples. Each data point represents the slope extracted from the linear regression in Figure 5. Error bars indicate the combined uncertainty from the regression and the averaging process. The dash-dotted line in (**a**) shows the weighted linear fit across all AA5456 slopes, with dashed bounds accounting for both measurement and fitting errors using root-sum-square.

To quantify the frequency dependency of the attenuation parameter, the frequency-attenuation relationship between 10 MHz and 16 MHz for each DoS level was fitted using linear regression to determine the frequency-attenuation slope. Figure 6 shows fitted slope values for the AA5456 and AA5052 samples. The error bars in Figure 6 represent the total uncertainty in the fitted slopes. This includes both the 95% confidence interval from the linear regression of frequency-dependent attenuation and the variability from averaging over multiple B-scan positions, calculated using root-sum-square (RSS) of the regression confidence interval and the averaging confidence interval (error bras in Figure 5). As shown in Figure 6a, the three AA5456 samples had frequency-attenuation slopes of 18.73, 20.77, and 18.96 dB/m per MHz, respectively, at the pristine state (DoS = 6.9 mg/cm^2^). As sensitization progresses to saturation (DoS = 39.9 mg/cm^2^), the slopes increase consistently across all samples, with total increments of 21.68, 21.08, and 20.09 dB/m per MHz, respectively. The 95% confidence intervals, represented by the error bars, are generally within ±6.96 dB/m per MHz and overlap across samples, demonstrating the consistency of this trend. A weighted linear regression was then applied to all AA5456 slope data. The resulting trend line is shown as a dot-dashed line, with dashed bounds representing the total uncertainty, calculated using RSS of the regression confidence interval and the total uncertainty in the fitted slopes. In contrast, the frequency-attenuation slopes obtained from the two AA5052 reference samples remain nearly constant over increasing heat treatment durations, as shown in Figure 6b. Figure 6 confirms the validity of the frequency-attenuation slope as a quantitative indicator of DoS progression. It is worth noting that the absolute slope values for AA5052 are larger than those of AA5456, and the variability between the two AA5052 samples is more pronounced compared to AA5456. This difference likely arises from intrinsic material properties such as composition, grain structure, and baseline attenuation behavior, which may be worth further investigation in future studies.

## 5. Conclusions

This study introduces the frequency-attenuation slope of longitudinal ultrasound waves as a quantitative index for characterizing sensitization in Al–Mg AA5456 alloys. The broadband responses of the sample plates were excited and acquired using an LUT system. A TFA technique was introduced to extract the frequency-dependent attenuation parameter of the pristine and sensitized samples. The slope of the frequency-attenuation relationship was found to correlate well with the DoS developed in heat-treated AA5456 samples. This study lays the foundation for future studies on exploring the frequency-attenuation slope for detecting other microstructural changes such as recrystallization and fatigue damage. The exact microstructural mechanisms underlying the increase in frequency-attenuation slope with sensitization, as well as the material-to-material differences observed, require further investigation involving detailed microstructural characterization. Additionally, characterizing the frequency dependency of the attenuation parameter could contribute to studying the fringe spectrum of ultrasound Fabry-Perot resonators for sensitization characterization in future studies [28].

## Data Availability

The data presented in this study are available on request from the corresponding author.

## Abbreviations

The following abbreviations are used in this manuscript:

Al–Mg	Aluminum–magnesium
ASTM	American society for testing and materials
DoS	Degree of sensitization
LUT	Laser ultrasonic testing
NAMLT	Nitric acid mass loss test
PP-FE	Primary pulse-first echo
PWAS	Piezoelectric wafer active sensors
TFA	Time-frequency analysis
UT	Ultrasonic testing
